# Allergic rhinitis in college students at Dongguan: a cross-sectional survey on disease burden, knowledge, and self-management

**DOI:** 10.3389/falgy.2025.1605074

**Published:** 2025-06-17

**Authors:** Xin Peng, Haiyi Yan, Haixin Feng, Shiqing Huang, Bingsong Zhang, Xueqin Huang

**Affiliations:** ^1^ School of Public Health, Guangdong Medical University, Dongguan, China; ^2^ Department of Otolaryngology, The First Dongguan Affiliated Hospital, Guangdong Medical University, Dongguan, China; ^3^ The Second Clinical Medical College, Guangdong Medical University, Dongguan, China

**Keywords:** allergic rhinitis, prevalence, environmental control, health education, life quality

## Abstract

This study examined the prevalence, clinical characteristics, disease knowledge, and quality of life impact of allergic rhinitis (AR) among college students in Dongguan, China. Using a customized questionnaire, we surveyed 1,531 participants (response rate: 85.1%) and identified an AR prevalence of 18.68% (95% CI: 16.72–20.63%). The study identified significant gaps in AR management, including underutilization of allergen testing (only 44.21% of AR patients underwent skin prick testing) and limited medication knowledge among 73% of participants. Environmental control measures were often neglected, and health education was inconsistently delivered, with 72.03% of students relying on the internet for AR information. The findings underscore the need for enhanced health education, improved access to diagnostic testing, and patient-centered communication strategies. Digital platforms and peer-led interventions are recommended to address these gaps and improve AR self-management.

## 1 Introduction

Allergic rhinitis (AR) is a non-infectious, IgE-mediated inflammatory disease of the nasal mucosa that occurs in atopic individuals upon allergen exposure. As a chronic condition, AR is primarily characterized by recurrent symptoms such as nasal discharge, sneezing, congestion, and itching, often accompanied by conjunctival irritation, ocular itching, or excessive tearing (1). In severe cases, it may lead to respiratory distress or anosmia.

Epidemiological studies indicate that AR affects approximately 10%–30% of adults and up to 40% of children in the U.S (2). In China, its prevalence has risen significantly from 11.1% to 17.6% over the past six years, with dust mites emerging as the predominant sensitizing aeroallergen in regions such as Hong Kong (3) and Guangdong Province (4).

The condition imposes a substantial burden on patients, manifesting as both physical discomfort—particularly during symptom flares—and impairments in daily activities and social functioning. These challenges can contribute to mental health disturbances in some individuals (5). Beyond Compromising quality of life, AR also represents a significant economic burden at the national level (6).

To better understand the prevalence and self-management status of AR among young adults and to provide actionable recommendations for health policy development, this cross-sectional study conducted a questionnaire survey among medical students at Guangdong Medical University in Dongguan, China. The study first estimated the prevalence of AR among college students and summarized disease-related behaviors, attitudes, and actions—factors that have rarely examined in prior research. Additionally, the study explored the association between the age of AR onset and self-management levels for the first time, revealing that a longer duration of the illness does not necessarily lead to improved self-management or knowledge of AR. This finding highlights a significant gap in public awareness, particularly among parents, and underscores the urgent need for increased education on AR for patients of all ages.

## 2 Materials and methods

### 2.1 Data

An online questionnaire survey was conducted among students at Guangdong Medical University in April 2023, coinciding with the peak AR incidence season in Guangdong Province. Using convenience sampling, the study recruited primarily undergraduate students, ranging from freshmen to seniors, along with a small number of fifth-year medical students. The survey was administered via a smartphone-based online platform and remained accessible for one month to maximize participation. A total of 1,800 questionnaires were distributed to students across all five academic years. After excluding incomplete or invalid responses, 1,531 fully completed questionnaires were retained for analysis, yielding a response rate of 85.1%. All collected responses underwent validation checks for consistency and completeness prior to inclusion in the dataset.

### 2.2 Methods

Considering the geographical factors that influence AR, we designed a questionnaire specifically tailored for participants in the Asia-Pacific region. The questionnaire comprised 45 questions covering various aspects, including basic personal information (e.g., gender, grade, and age at disease diagnosis), disease-related details (e.g., allergens, AR attack patterns and frequency, complications, and disease awareness), as well as general self-efficacy and nose-related quality of life. The complete questionnaire has been translated into English and is provided as a Supplementary File. Data description and analysis were conducted after the survey was completed.

All participants were informed about the purpose and process of the AR survey and provided their written consent to report the survey results. This study was approved by the Medical Ethics Review Committee of the First Dongguan Affiliated Hospital, Guangdong Medical University.

### 2.3 Statistical analysis

The overall AR prevalence was estimated at 17.6% (±3%), based on published literature (1, 7). A sample size of 1,800 patients was determined to provide 90% power to detect the true prevalence (*α*=0.05), accounting for a 10% non-response rate.

The prevalence of AR and its 95% confidence interval were estimated using the maximum likelihood estimator. For AR patients, categorical variables were presented as frequencies and percentages (%), and comparisons between groups were made using the chi-square test. Continuous data were described using means and standard deviations, with analysis of variance (ANOVA) employed for comparisons between multiple groups. To explore the association between the age of AR onset and self-management status, participants were categorized into four groups based on the age at diagnosis: diagnosed in primary school (<12 years old), middle school (12–16 years old), high school (16–18 years old), and college (>18 years old).

All statistical analyses were conducted using SAS v9.4 (SAS Institute, Inc., Cary, North Carolina). A two-sided *P*-value of <0.05 was considered statistically significant.

## 3 Results

### 3.1 Characteristics of the study patients and AR prevalence

We distributed 1,800 questionnaires and received 1,531 valid responses, achieving an 85.1% response rate. Table 1 presents the characteristics of the study participants. A total of 286 out of 1,531 respondents reported having AR, resulting in a prevalence rate of 18.68% (95% CI: 16.72–20.63%). Subgroup analysis revealed a prevalence of 19.17% among males and 18.33% among females, with no significant gender difference observed (*χ*^2^ = 0.17, *P* = 0.6772) (Supplementary Material, Table S1).

**Table 1:** Characteristics of the study patients (*n* = 286).

Characteristics	Age of diagnosed			
	Primary school (*n* = 87)	Middle school (*n* = 60)	High school (*n* = 78)	College (*n* = 61)
Sex, *n* (%)				
Female	43 (49.43)	33 (55.00)	51 (65.38)	35 (57.38)
Male	44 (50.57)	27 (45.00)	27 (34.62)	26 (42.62)
Temporal pattern, *n* (%)				
Seasonal	26 (29.89)	24 (40.00)	33 (42.31)	24 (39.34)
Perennial	39 (44.83)	21 (35.00)	20 (25.64)	21 (34.43)
Episodic	22 (25.29)	15 (25.00)	25 (32.05)	16 (26.23)
Symptom frequency, *n* (%)				
Intermittent	59 (67.82)	38 (63.33)	63 (80.77)	39 (63.93)
Persistent	28 (32.18)	22 (36.67)	15 (19.23)	22 (36.07)
Severity				
Mild	61 (70.11)	36 (60.00)	61 (78.21)	47 (77.05)
Moderate-severe	26 (29.89)	24 (40.00)	17 (21.79)	14 (22.95)
Common comorbidity, *n* (%)				
Asthma	13 (14.94)	4 (6.67)	4 (5.13)	6 (9.84)
Sinusitis	11 (12.64)	12 (20)	17 (21.79)	11 (18.03)
Pharyngitis	6 (6.9)	6 (10)	7 (8.97)	8 (13.11)
Conjunctivitis	10 (11.49)	4 (6.67)	5 (6.41)	6 (9.84)

Based on responses to Question 2 (“What's your current grade?”) and Question 4 (“How many years have you been diagnosed with AR?”), we estimated the onset age of AR and categorized participants into four groups as described in Section 2.3. The proportions of each group were as follows: 30.42% for those diagnosed in primary school or earlier, 20.98% for middle school, 27.27% for high school, and 21.33% for college. The average duration of AR was approximately 7 years, with a maximum duration of 20 years and a minimum of 1 year. A detailed statistical description of the questionnaire is provided in the Supplementary File.

### 3.2 Clinical features

#### 3.2.1 AR attacking features

**Figure 1:**
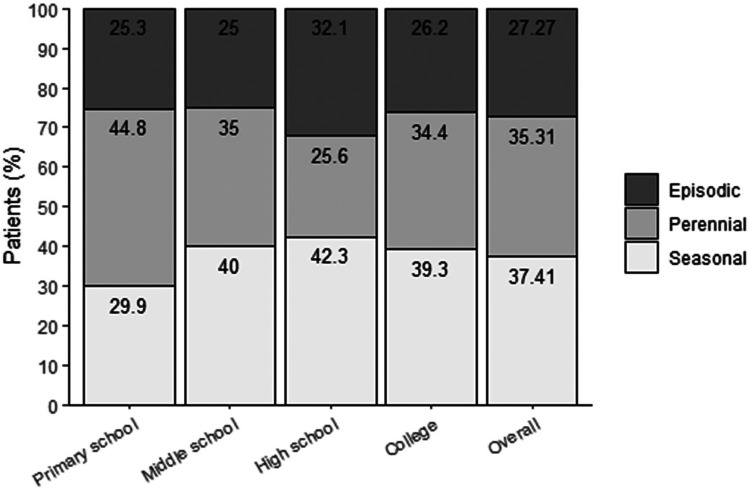
Disease attacking type divided by temporal pattern.

In this study, seasonal attacks were the most common, followed by perennial attacks and episodic attacks, as shown in Figure 1. Patients with intermittent attacks predominantly experienced mild symptoms, while a higher proportion of patients with persistent attacks reported moderate to severe symptoms rather than mild ones (Supplementary Material, Table S2). A statistically significant difference in symptom severity was observed across different types of AR (*χ*^2^ = 48.29, *P* < 0.0001), suggesting that, among college students with AR in Dongguan City, those with intermittent attacks tend to experience milder symptoms compared to those with persistent attacks.

#### 3.2.2 Allergen detection and allergen type composition

**Figure 2:**
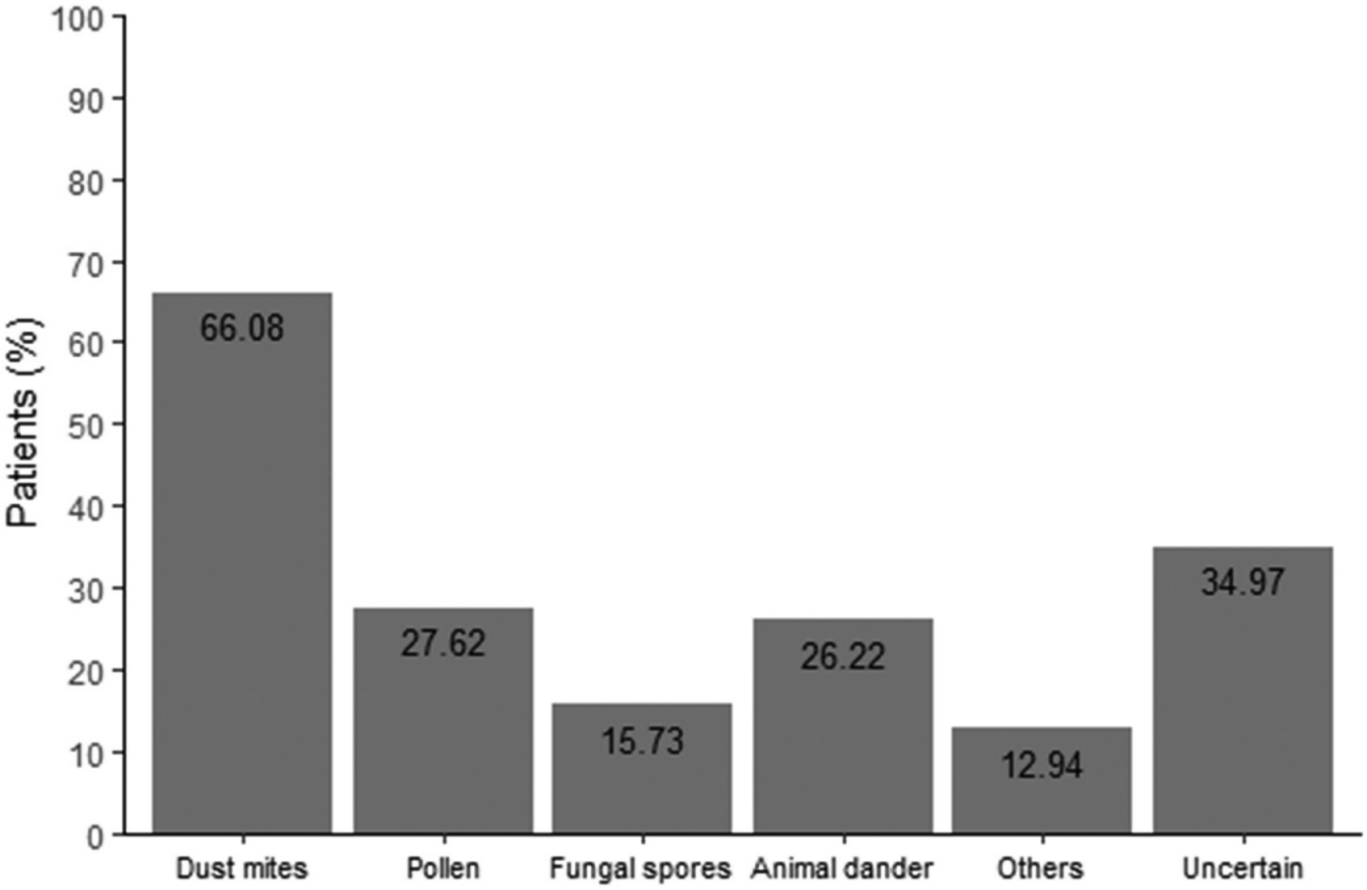
Types of allergens in college students.

The survey results revealed that, among the 286 patients, 190 were diagnosed based on nasal cavity examination and medical history at a general hospital, though allergen testing was not conducted. In contrast, 84 patients underwent allergen testing, representing a testing rate of 44.21%. Among those who were tested, dust mites emerged as the primary allergen, as illustrated in Figure 2.

Additionally, patients diagnosed with AR after college were more likely to undergo allergen testing compared to those diagnosed before college (*χ*^2^ = 7.35, *P* = 0.0067). This suggests that patients diagnosed earlier in life tend to have clearly identified allergens (Supplementary Material, Table S3).

#### 3.2.3 AR symptoms

Among the 286 patients, the most common symptoms of AR were continuous episodes of paroxysmal sneezing (82.87%), watery nasal discharge (79.02%), nasal congestion (75.17%), nasal itching (65.73%), and itchy or red eyes (37.06%). Less common symptoms included hyposmia (25.87%), cough (14.69%), and chest tightness (6.29%). The most frequent complications were chronic sinusitis (17.83%), followed by asthma (9.44%), allergic pharyngitis (9.44%), nasal polyps (8.74%), and conjunctivitis (8.74%).

#### 3.2.4 Treatment usage and knowledge

In this study, 146 out of 286 respondents reported not visiting any medical institution for AR in the past year, while the remaining 140 had at least one medical visit. The results indicated that college-age AR patients tend to have insufficient medical consultations for managing their condition. A significant difference in medical visits was observed between patients diagnosed before and after college (*χ*^2^ = 12.29, *P* = 0.0005). As shown in Supplementary Material Table S4, patients diagnosed at an older age were more likely to visit doctors regularly, which may explain why this group typically experiences milder symptoms. In contrast, younger patients, having dealt with the disease for a longer period, may experience mental and physical fatigue, leading to reluctance in seeking proper management.

**Figure 3:**
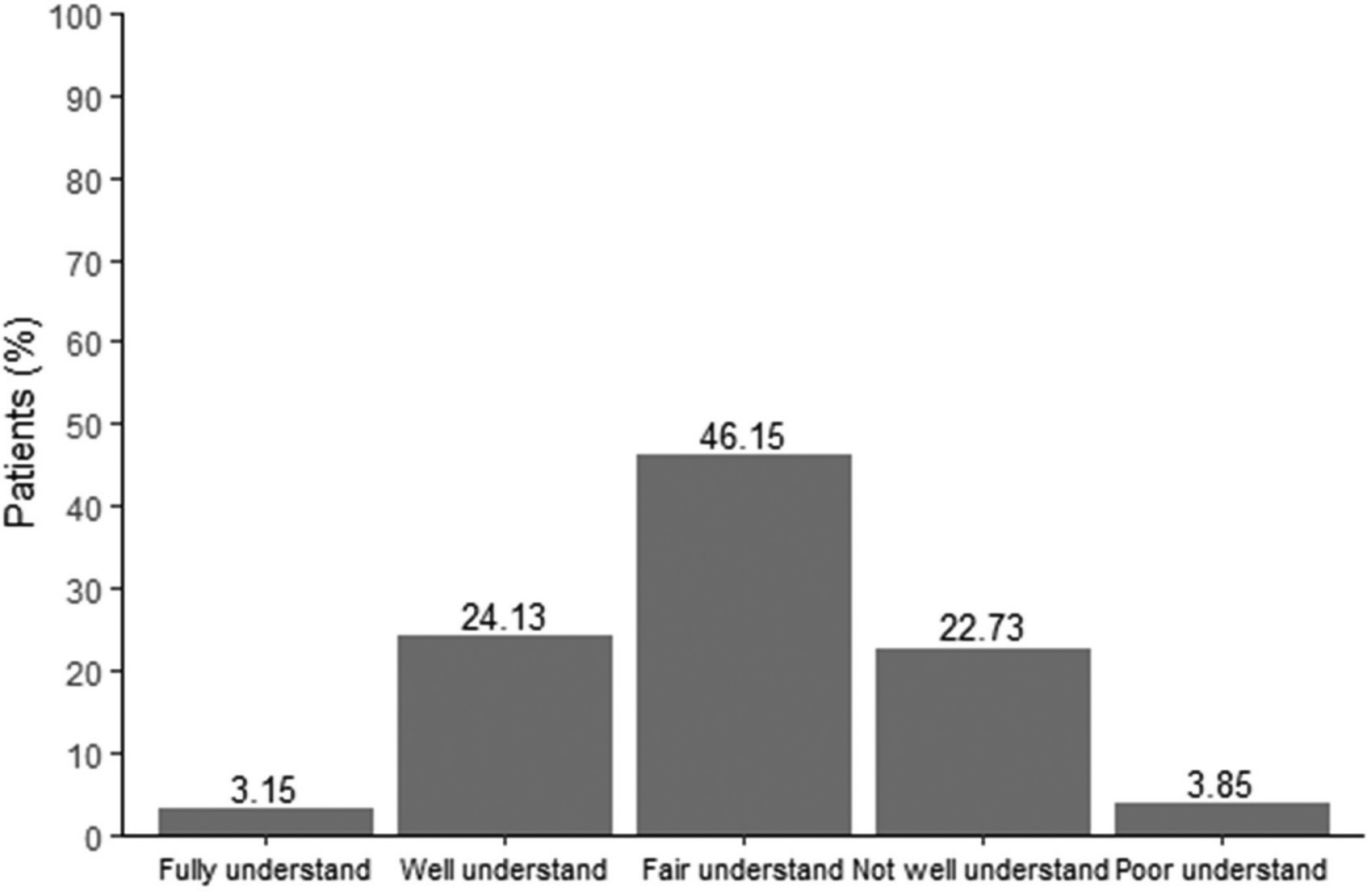
Understanding the correct use of drugs to treat AR.

**Figure 4:**
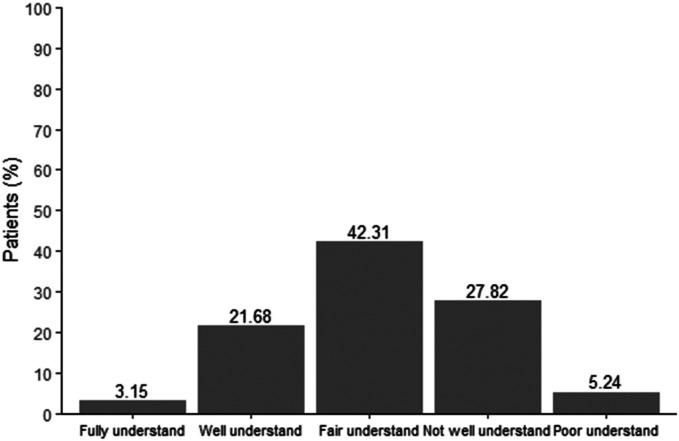
Understanding the frequency of drug use to treat AR.

Regarding knowledge of AR, including allergens, medication usage, and disease management, the majority of patients demonstrated a medium to low level of understanding, despite suffering from the condition for an extended period. As shown in Figure 3, 27.28% of patients had limited or inadequate knowledge about the correct use of medications, while Figure 4 revealed that only 24.83% of patients had a solid understanding of the frequency of medication use.

### 3.3 Cognitive level and needs

Self-efficacy refers to an individual's self-perceived ability to effectively handle various situations. In this survey, the mean score for GSE was 28.63 out of 40, and the mean score for confidence in AR management was 6.33 out of 10, suggesting that patients generally have a strong belief in their ability to manage their health condition effectively. The means and medians for each item are presented in Table 2.

**Table 2:** General self-efficacy scale (GSES) items and item mean, median.

Item	Mean	Median
1: I can manage to solve difficult problems if I try hard enough.	3.18	3
2: If someone opposes me, I can find the means and ways to get what I want.	3.03	3
3: It is easy for me to stick to my aims and accomplish my goals.	2.70	3
4: I am confident I can deal efficiently with unexpected events.	2.71	3
5: Thanks to my talents and skills, I know how to handle unexpected situations.	2.70	3
6: I can solve most problems if I try hard enough.	3.01	3
7: I stay calm when facing difficulties because I can handle them.	2.88	3
8: I stay calm when facing difficulties because I can handle them.	2.79	3
9: If I am in trouble, I can think of a solution.	2.96	3
10: I can handle whatever comes my way.	2.67	3

In this study, AR patients primarily acquire health education through the Internet (72.03%), followed by doctor visits (51.05%). The most sought-after information among AR patients includes new treatments (68.18%), symptom control strategies (69.58%), and daily prevention methods (65.38%).

### 3.4 Impact on quality of life

The results revealed that nasal symptoms significantly disrupted the daily lives of college students. The most commonly affected activities included entering and exiting air-conditioned rooms (44.06%), reading (31.47%), doing housework (30.77%), exercising (27.97%), and being in smoking environments (27.97%). In this survey, the degree of distress caused by AR in various aspects—such as sleep, nasal symptoms, eye symptoms, non-nasal/eye symptoms, and emotional status—was rated on a scale from 0 (no trouble) to 6 (extremely troubled). The findings showed that sleep disturbances in college students were primarily due to difficulty falling asleep (average score: 2.15). Nasal symptoms were mostly characterized by sneezing (average score: 3.42), while eye symptoms were mainly eye itching (average score: 2.07). The most significant non-nasal/eye symptom was fatigue (average score: 2.56), and the emotional concern was inner impatience or restlessness (average score: 1.93). Interestingly, college students reported being more troubled by the need to blow their noses repeatedly due to nasal symptoms (mean score: 3.42) than by other related issues, such as rubbing their noses or eyes, or carrying tissues or handkerchiefs.

### 3.5 Control of AR

We collected data on patients' symptomatic episodes over the past four weeks. The survey results revealed that only 3.50% of college student AR patients experienced no symptoms during this period. Among those who had symptoms, the proportions of patients experiencing nasal congestion, sneezing, nasal itching, and runny nose almost every day in the past four weeks were 21.68%, 19.93%, 20.28%, and 19.93%, respectively. Additionally, 40.21% of AR patients reported an increase in medication use due to allergic respiratory diseases during the past four weeks.

## 4 Discussion

This cross-sectional survey at Guangdong Medical University in Dongguan, China, revealed an AR prevalence of 18.68% (95% CI: 16.72–20.63%) among college students, closely aligning with the national prevalence of 17.6% for Chinese adults (*p* > 0.05). This consistency underscores AR as a significant public health issue among young adults in Dongguan, comparable to broader Chinese populations. However, our findings show a slightly lower prevalence compared to a China-wide meta-analysis reporting 19% (95% CI: 14%–25%) for adults and 22% (95% CI: 17%–27%) for pediatric populations (8). These discrepancies may stem from differences in diagnostic methodologies, participant demographics, or regional allergen exposures, highlighting the need for careful interpretation of the findings.

A notable finding is the underutilization of allergen testing, with only 44.21% of AR patients having undergone skin prick testing (SPT) or other diagnostic assays. Dust mites were the predominant allergen among tested participants, consistent with prior studies in subtropical regions like Guangdong (4, 9). Patients diagnosed earlier in life tend to have clearly identified allergens. The low testing rate may reflect limited awareness among healthcare providers and patients about the diagnostic value of SPT, which is critical for identifying specific allergens and tailoring environmental control measures. On the other hand, improvement in medical conditions also influence patients' willingness to undergo SPT (10). Although the testing methods remain unchanged (still using skin prick tests or blood tests for allergens), there have been advancements in reagents (11). However, it should be noted that the diagnosis of AR does not rely solely on laboratory results, but rather requires both clinical manifestations and laboratory findings for confirmation. Increasing access to SPT and educating patients about its role in AR management could enhance allergen avoidance strategies, thereby reducing symptom burden and improving quality of life.

The study highlights significant gaps in AR self-management, particularly in medication knowledge. Approximately 73% of participants demonstrated limited understanding of proper medication use and frequency, despite many having managed AR for extended periods. This issue was particularly evident among those with longer disease durations, who exhibited reduced healthcare engagement and treatment adherence. Such trends may stem from patient fatigue or dissatisfaction with recurrent symptoms and perceived treatment inefficacy, as noted in prior research (12). To address this, healthcare providers should adopt patient-centered communication strategies, using clear, jargon-free language to explain treatment regimens and emphasize the importance of consistent medication adherence.

Environmental control and health education are critical yet underemphasized components of AR management (13). Our findings indicate that environmental control measures are often neglected, despite their foundational role in reducing allergen exposure. Additionally, health education is inconsistently delivered, with 72.03% of students relying on the internet for AR information. Integrating digital platforms into educational interventions could enhance reach and engagement, particularly among young adults (14). Universities could develop multimedia campaigns, such as short-form videos on platforms like Douyin, to provide evidence-based guidance on AR prevention, symptom control, and treatment adherence. Such initiatives could shift the paradigm from reactive treatment to proactive prevention, fostering sustained disease management.

Moderate self-efficacy scores (mean GSE: 28.63/40; AR-specific confidence: 6.33/10) suggest that students have a baseline confidence in managing AR, but targeted interventions could further strengthen their capabilities. Peer-led workshops or mobile health applications could empower students with practical skills and reinforce self-management behaviors. The substantial impact of AR on quality of life-evidenced by sleep disturbances, academic disruptions, and fatigue—underscores the need for holistic management strategies that address both physical and psychosocial burdens.

Limitations of this study include potential selection bias, as participants were medical students with likely higher health literacy than the general population, which may overestimate AR awareness and management practices. Additionally, the absence of pre-university geographical data may obscure regional variations in allergen sensitization, given Dongguan's unique environmental profile. Future studies should recruit diverse populations and incorporate longitudinal designs to better understand AR's long-term impacts and management trends.

## 5 Conclusion

The findings underscore the significant prevalence and burden of AR among college students in Dongguan, coupled with substantial gaps in disease knowledge and self-management. Comprehensive health education programs, leveraging digital platforms and community-based interventions, are essential to enhance AR awareness, optimize treatment adherence, and improve quality of life. Policymakers and educators should prioritize integrating AR education into university health services to address this growing public health challenge.

## Data Availability

The datasets presented in this article are not readily available because the datasets supporting this study are available from the corresponding author upon reasonable request. Requests to access the datasets should be directed to Xueqin Huang, huangxueqin@gdmu.edu.cn.
