# Supplementary material for: Allergic rhinitis in college students at Dongguan: a cross-sectional survey on disease burden, knowledge, and self-management
Source: Front Allergy. 2025 Jun 17;6:1605074. doi: 10.3389/falgy.2025.1605074 (PMC12209292; doi:10.3389/falgy.2025.1605074)
Supplement: Supplementary file 1 [file Table2.doc]

# Allergic Rhinitis Questionnaire

Part 1. General information

1. Are you:

- Male
- Female

2. What year of university are you in?

- First year
- Third year
- Fifth year
- Second year
- Fourth year

3. Have you been definitively diagnosed with allergic rhinitis by a specialist healthcare provider?

- Yes
- No

Part 2. Disease-related information

4. How many years have you been diagnosed with allergic rhinitis? __________

5. Do you know about allergic rhinitis?

- Yes
- A little
- No

6. Which of the following matches your condition?

- Seasonal attacks
- Episodic attacks
- Perennial attacks

7. What's the frequency of your attacks?

- Intermittent attacks (symptom attacks < 4 days/week or < 4 consecutive weeks)
- Persistent attacks (symptom attacks ≥ 4 consecutive weeks)

8. What's the severity of the disease?

- Mild
- Moderate - severe

9. How were you diagnosed with allergic rhinitis?

- By inquiring about the medical history and examining the nasal cavity
- By allergen testing (such as skin prick test, blood test for IgE antigen detection, etc.)
- Others

10. Your current known allergens include (multiple choices allowed):

- Dust mites
- Fungal spores
- Pollen
- Animal dander
- Others
- Uncertain

11. What are your current symptoms related to allergic rhinitis (multiple choices allowed)?

- Continuous episodes of paroxysmal sneezing
- Watery nasal discharge
- Nasal congestion
- Nasal itching
- Itchy eyes, red eyes
- Hyposmia
- Cough
- Chest tightness
- No related symptoms

12. Do you have the following comorbidities (multiple choices allowed)?

- Chronic sinusitis
- Asthma
- Allergic pharyngitis
- Nasal polyps
- Conjunctivitis
- Upper respiratory cough syndrome
- Secretory otitis media
- Atopic dermatitis
- Others
- No comorbidities

13. Have you ever worried that drugs for allergic rhinitis will cause relevant side effects?

- Yes
- No

14. Have you ever had the following adverse drug reactions (multiple choices allowed)?

- Nasal dryness and bleeding
- Osteoporosis
- Others
- Decreased immunity
- Hypertension, arteriosclerosis
- No adverse drug reactions

15. Do you know the correct use of allergic rhinitis drugs?

- Fully understand
- Fair understand
- Poor understand
- Well understand
- Not well understand

16. Do you know the frequency of use of allergic rhinitis drugs?

- Fully understand
- Well understand
- Fair understand
- Poor understand
- Not well understand

17. After medication, have you ever felt that the drug has strong irritation?

- Yes
- No

18. Can you afford to treat allergic rhinitis?

- I can definitely afford it.
- I can't really afford it.
- I can somewhat afford it.
- I definitely can't afford it.

19. The number of outpatient visits due to allergic rhinitis in the past year is ____ times.

20. Your expenditure for the treatment of allergic rhinitis in the past year is about ____ yuan.

21. If you give a score, what do you think of your medication compliance performance for allergic rhinitis? ____ (0 points cannot fully comply, 10 points fully comply)

22. If you give a score, what is your confidence in doing a good job in the management of allergic rhinitis? ____ (0 points: no confidence at all, 10 points: full confidence)

23. If you give a score, how much do you think you know about allergic rhinitis? ____ (0 points totally don't understand, 10 points totally understand)

24. From which channels did you learn about relevant health education knowledge (multiple choices allowed)?

- Internet
- Community lecture
- Health education for nurses
- Other
- Television
- Doctor visit
- Hospital Brochure
- No channels

25. What do you want to know more about allergic rhinitis (multiple choices allowed)?

- New treatment
- Etiology
- Daily prevention methods
- Medication method
- Other
- Drug side effects
- Symptom control methods
- Psychological adjustment skills
- Relationship with other diseases (such as asthma, COPD, conjunctivitis)

Part 3. General Self-efficacy

Note: self-efficacy refers to the belief that individuals can take appropriate actions to face environmental challenges, which is what we call confidence. Please comment on your confidence in the management of allergic rhinitis and put "√" in the option box.

26. I can always manage to solve difficult problems if l try hard enough.

- Not at all true (1 point)
- Barely true (2 point)
- Moderately true (3 point)
- Exactly true (4 point)

27. If someone opposes me, I can find means and ways to get what I want.

- Not at all true (1 point)
- Barely true (2 point)
- Moderately true (3 point)
- Exactly true (4 point)

28. It is easy for me to stick to my aims and accomplish my goals.

- Not at all true (1 point)
- Barely true (2 point)
- Moderately true (3 point)
- Exactly true (4 point)

29. I am confident that I could deal efficiently with unexpected events

- Not at all true (1 point)
- Barely true (2 point)
- Moderately true (3 point)
- Exactly true (4 point)

30. Thanks to my resourcefulness, l know how to handle unforeseen situations.

- Not at all true (1 point)
- Barely true (2 point)
- Moderately true (3 point)
- Exactly true (4 point)

31. I can solve most problems if l invest the necessary effort.

- Not at all true (1 point)
- Barely true (2 point)
- Moderately true (3 point)
- Exactly true (4 point)

32. I can remain calm when facing difficulties because I can rely on my coping abilities.

- Not at all true (1 point)
- Barely true (2 point)
- Moderately true (3 point)
- Exactly true (4 point)

33. When I am confronted with a problem, I can usually find several solutions.

- Not at all true (1 point)
- Barely true (2 point)
- Moderately true (3 point)
- Exactly true (4 point)

34. If I am in a bind, I can usually think of something to do.

- Not at all true (1 point)
- Barely true (2 point)
- Moderately true (3 point)
- Exactly true (4 point)

35. No matter what comes my way, I’m usually able to handle it.

- Not at all true (1 point)
- Barely true (2 point)
- Moderately true (3 point)
- Exactly true (4 point)

36. I can complete the management of allergic rhinitis. ____ (0-10 points)

Part 4. Impact on quality of life

37. You can recall the situation that your nasal symptoms have caused trouble to your life. Below is a list of activities that are limited by nasal symptoms. We have listed some activities, hoping to help you find out the three main activities that you have been restricted due to your nasal symptoms in the past two weeks. Please tick "√" on the items that meet the requirements and evaluate the impact of this option. (Multiple choices allowed)

- Ride a bicycle
- Reading
- Shopping
- Do home repair
- Do housework
- Access to air-conditioned rooms
- Watch TV
- Exercise or exercise
- Morning exercises
- Using the computer
- Table tennis
- Play with pets
- Play with your daughters or grandchildren
- Participate in group sports
- Driving
- Singing
- Conduct normal social activities
- Sex life
- Badminton
- Chat
- Eating
- Use vacuum cleaner
- Visit friends or relatives
- Go out for a walk
- Taking children to and from school
- Outdoor activities
- Work
- Sitting outdoors
- Take children to the park
- Exposure to smoking environment
- Others

38. Sleep situation

| Item | Not at all troublesome | Barely troublesome | Somewhat troublesome | Moderately troublesome | Very troublesome | Quite troublesome | Extremely troublesome |
| --- | --- | --- | --- | --- | --- | --- | --- |
| Falling asleep |  |  |  |  |  |  |  |
| Wake up at night |  |  |  |  |  |  |  |
| Poor sleep at night |  |  |  |  |  |  |  |

39. Practical problem

| Item | Not at all troublesome | Barely troublesome | Somewhat troublesome | Moderately troublesome | Very troublesome | Quite troublesome | Extremely troublesome |
| --- | --- | --- | --- | --- | --- | --- | --- |
| Inconvenient due to having to bring paper towels or handkerchiefs |  |  |  |  |  |  |  |
| Need to rub your nose / eyes |  |  |  |  |  |  |  |
| Need to blow your nose repeatedly |  |  |  |  |  |  |  |

40. Non nasal / ocular symptoms

| Item | Not at all troublesome | Barely troublesome | Somewhat troublesome | Moderately troublesome | Very troublesome | Quite troublesome | Extremely troublesome |
| --- | --- | --- | --- | --- | --- | --- | --- |
| Lack of energy |  |  |  |  |  |  |  |
| Thirsty |  |  |  |  |  |  |  |
| Reduced working ability repeatedly |  |  |  |  |  |  |  |
| Tired |  |  |  |  |  |  |  |
| Difficulty concentrating |  |  |  |  |  |  |  |
| Headache |  |  |  |  |  |  |  |
| Exhausted |  |  |  |  |  |  |  |

41. Nasal symptoms

| Item | Not at all troublesome | Barely troublesome | Somewhat troublesome | Moderately troublesome | Very troublesome | Quite troublesome | Extremely troublesome |
| --- | --- | --- | --- | --- | --- | --- | --- |
| Stuffy nose |  |  |  |  |  |  |  |
| Runny nose |  |  |  |  |  |  |  |
| Sneeze |  |  |  |  |  |  |  |
| Nasal discharge flows back to the throat |  |  |  |  |  |  |  |
| Stuffy nose |  |  |  |  |  |  |  |

42. Ocular symptoms

| Item | Not at all troublesome | Barely troublesome | Somewhat troublesome | Moderately troublesome | Very troublesome | Quite troublesome | Extremely troublesome |
| --- | --- | --- | --- | --- | --- | --- | --- |
| Itchy eye |  |  |  |  |  |  |  |
| Tears |  |  |  |  |  |  |  |
| Ocular pain |  |  |  |  |  |  |  |
| Ocular swelling |  |  |  |  |  |  |  |

43. Emotion

| Item | Not at all troublesome | Barely troublesome | Somewhat troublesome | Moderately troublesome | Very troublesome | Quite troublesome | Extremely troublesome |
| --- | --- | --- | --- | --- | --- | --- | --- |
| Depressed |  |  |  |  |  |  |  |
| Internal impatience or restlessness |  |  |  |  |  |  |  |
| Easily irritated |  |  |  |  |  |  |  |
| Embarrassed by symptoms |  |  |  |  |  |  |  |

Part 5. Control of allergic rhinitis

44. Please tick "√" on the matching options. How many times have you suffered from allergic rhinitis in the past 4 weeks?

| Item | Never | 2 days a week at most | More than 2 days a week | Almost every day |
| --- | --- | --- | --- | --- |
| 1. Nasal congestion |  |  |  |  |
| 2. Sneeze |  |  |  |  |
| 3. Itchy nose |  |  |  |  |
| 4. Runny nose |  |  |  |  |
| 5. Shortness of breath / dyspnea |  |  |  |  |
| 6. Chest treble / wheeze |  |  |  |  |
| 7. Chest tightness during exercise |  |  |  |  |
| 8. Tired / having difficulty doing housework every day |  |  |  |  |
| 9. Wake up in the middle of the night |  |  |  |  |

45. How many times have you been in the past 4 weeks:

| Item | I won't take any medicine | Never | Less than 7 days | 7 days or more |
| --- | --- | --- | --- | --- |
| Increase the use of drugs due to allergic respiratory diseases (asthma, rhinitis and allergy)? |  |  |  |  |
