# Supplementary material for: Allergic rhinitis in college students at Dongguan: a cross-sectional survey on disease burden, knowledge, and self-management
Source: Front Allergy. 2025 Jun 17;6:1605074. doi: 10.3389/falgy.2025.1605074 (PMC12209292; doi:10.3389/falgy.2025.1605074)
Supplement: Supplementary file 2 [file Table3.docx]

**Table** **S1 Comparison of the prevalence of AR between male and female**

| Sex | Participant with AR | Participant without AR | Total | *P* |
| --- | --- | --- | --- | --- |
| Male | 124 (19.17%) | 523 (80.83%) | 647 | 0.6772 |
| Female | 162 (18.33%) | 722 (81.67%) | 884 |  |
| Total | 286 | 1245 | 1531 |  |

**Table S2 Comparison of severity between different types of AR**

| Types of AR | Mild | Moderate-severe | Total | *P* |
| --- | --- | --- | --- | --- |
| Intermittent | 167 (83.92%) | 32 (16.08%) | 199 | <0.0001 |
| Persistent | 38 (43.68%) | 49 (56.32%) | 87 |  |
| Total | 205 | 81 | 286 |  |

**Table S3 Comparison of the presence of known allergens at the age of diagnosis for AR**

| Age of  diagnosis | Are there any known allergens | | | *P* |
| --- | --- | --- | --- | --- |
|  | Yes | No | Total |  |
| Under college age | 172 (76.44%) | 53 (23.56%) | 225 | 0.0067 |
| College age | 36 (59.02%) | 25 (40.98%) | 61 |  |
| Total | 208 | 78 | 286 |  |

**Table S4 General Self-Efficacy Scale (GSES) items and item mean, median**

| Item | Mean | Median |
| --- | --- | --- |
| 1: I can manage to solve difficult problems if I try hard enough. | 3.18 | 3 |
| 2: If someone opposes me, I can find the means and ways to get what I want. | 3.03 | 3 |
| 3: It is easy for me to stick to my aims and accomplish my goals. | 2.70 | 3 |
| 4: I am confident I can deal efficiently with unexpected events. | 2.71 | 3 |
| 5: Thanks to my talents and skills, I know how to handle unexpected situations. | 2.70 | 3 |
| 6: I can solve most problems if I try hard enough. | 3.01 | 3 |
| 7: I stay calm when facing difficulties because I can handle them. | 2.88 | 3 |
| 8: I stay calm when facing difficulties because I can handle them. | 2.79 | 3 |
| 9: If I am in trouble, I can think of a solution. | 2.96 | 3 |
| 10: I can handle whatever comes my way. | 2.67 | 3 |
